# Suppression of Aggrus/podoplanin-induced platelet aggregation and pulmonary metastasis by a single-chain antibody variable region fragment

**DOI:** 10.1002/cam4.320

**Published:** 2014-08-16

**Authors:** Kenichi Miyata, Satoshi Takagi, Shigeo Sato, Hiroshi Morioka, Kiyotaka Shiba, Tamiko Minamisawa, Miho Takami, Naoya Fujita

**Affiliations:** 1 Division of Experimental Chemotherapy, The Cancer Chemotherapy Center, Japanese Foundation for Cancer Research3-8-31, Ariake, Koto-ku, Tokyo, 135-8550, Japan; 2 Department of Medical Genome Sciences, Graduate School of Frontier Sciences, The University of Tokyo5-1-5, Kashiwanoha, Kashiwa-shi, Chiba, 277-8561, Japan; 3 Graduate School of Pharmaceutical Sciences, Kumamoto University5-1 Oe-honmachi, Kumamoto, 862-0973, Japan; 4 Division of Protein Engineering, The Cancer Institute, Japanese Foundation for Cancer Research3-8-31, Ariake, Koto-ku, Tokyo, 135-8550, Japan

**Keywords:** Aggrus/podoplanin, phage display, platelet aggregation, scFv, tumor metastasis

## Abstract

Almost all highly metastatic tumor cells possess high platelet aggregating abilities, thereby form large tumor cell-platelet aggregates in the microvasculature. Embolization of tumor cells in the microvasculature is considered to be the first step in metastasis to distant organs. We previously identified the platelet aggregation-inducing factor expressed on the surfaces of highly metastatic tumor cells and named as Aggrus. Aggrus was observed to be identical to the marker protein podoplanin (alternative names, T1*α*, OTS-8, and others). Aggrus is frequently overexpressed in several types of tumors and enhances platelet aggregation by interacting with the platelet receptor C-type lectin-like receptor 2 (CLEC-2). Here, we generated a novel single-chain antibody variable region fragment (scFv) by linking the variable regions of heavy and light chains of the neutralizing anti-human Aggrus monoclonal antibody MS-1 with a flexible peptide linker. Unfortunately, the generated KM10 scFv failed to suppress Aggrus-induced platelet aggregation in vitro. Therefore, we performed phage display screening and finally obtained a high-affinity scFv, K-11. K-11 scFv was able to suppress Aggrus-induced platelet aggregation in vitro. Moreover, K-11 scFv prevented the formation of pulmonary metastasis in vivo. These results suggest that K-11 scFv may be useful as metastasis inhibitory scFv and is expected to aid in the development of preclinical and clinical examinations of Aggrus-targeted cancer therapies.

## Introduction

Metastasis is the major cause of cancer-related mortality, accounting for approximately 90% of cancer deaths [1]. Because the process of metastasis is complicated, an optimal treatment strategy remains uncertain. It is well recognized that the interaction between platelets and tumor cells plays an important role in successful hematogenous metastasis [2, 3]. The main function of platelets under physiological conditions is to stop the bleeding after tissue trauma and vascular injury [2, 4]. However, under pathological conditions, platelets enhance survival of tumor cells by protecting them from immunological assault within the circulation or from shear stress [2]. Reportedly, antiplatelet or anticoagulant drugs extend progression-free survival in patients with progressive cancer [5, 6]. However, it is impossible to administrate such antiplatelet or anticoagulant drugs to some cancer patients with thrombocytopenia induced by antitumor drug treatment [5].

Aggrus is a type I transmembrane sialoglycoprotein that can induce platelet aggregation by directly binding to C-type lectin-like receptor 2 (CLEC-2) expressed on the platelet surface [5, 7]. Although Aggrus was originally reported as a specific lymphatic endothelial marker protein called podoplanin, it is expressed by various malignant tumors, including squamous cell carcinomas, mesotheliomas, glioblastomas, and osteosarcomas [5, 8]. Therefore, Aggrus is now considered unsuitable as a lymphatic endothelial marker. Moreover, Aggrus overexpression is reportedly correlated with poor prognosis in oral cancers [9]. Because Aggrus can facilitate hematogenous metastasis, it presents a promising therapeutic target for antitumor and antimetastatic agents [5]. Recently, we successfully generated a mouse anti-human Aggrus monoclonal antibody (mAb) named MS-1. MS-1 mAb effectively suppressed Aggrus-induced platelet aggregation and Aggrus-mediated pulmonary metastasis [10].

In clinical, antibodies exhibit higher specificity to targeted proteins, have longer activities, and produce fewer side effects than traditional chemotherapeutic agents. There are currently 13 antibodies approved by the US Food and Drug Administration for various cancers and several others are currently under clinical trial [11, 12]. Despite high effectiveness, the costs of manufacturing antibodies are high because of the requirement of a mammalian expression system [13, 14]. Converting antibodies into small recombinant antibodies, including single-chain antibody variable region fragments (scFvs), may help to lower the high cost of production [15]. The scFv is the smallest unit of an immunoglobulin molecule with function in antigen-binding activities and consists of variable regions of the heavy chain (V_H_), variable regions of the light chain (V_L_), and a flexible peptide linker [16, 17]. The scFv can be produced by bacterial expression systems (e.g., *Escherichia coli* [*E. coli*]), thereby reducing the manufacturing cost [13]. Furthermore, the affinity or activity of scFv can be easily amendable by genetic manipulation [17]. Therefore, we attempted to convert MS-1 mAb into scFv.

We successfully generated a novel scFv by linking V_H_ and V_L_ of mouse mAb MS-1 with a flexible peptide linker. The generated KM10 scFv retained affinity for Aggrus. However, the affinity of KM10 scFv for Aggrus is lower than that of MS-1 mAb. To improve affinity to Aggrus, we performed phage display screening. The affinity-maturated K-11 scFv exhibited improved affinity and suppressed Aggrus-induced platelet aggregation in vitro and Aggrus-mediated pulmonary metastasis in vivo. Thus, K-11 scFv presents a useful tool to aid in the development of Aggrus-targeted metastasis inhibitors.

## Materials and Methods

### Cell lines

CHO and H226 cells were purchased from American Type Culture Collection (ATCC) and cultured in RPMI 1640 media (Wako, Osaka, Japan) supplemented with 10% fetal bovine serum (FBS). PC-10 (Immuno-Biological Laboratories, Gunma, Japan) cells were cultured in RPMI 1640 media supplemented with 10% FBS. Aggrus-transfected CHO cells were cultured as previously described [10].

### Plasmid construction

**Figure 1:**
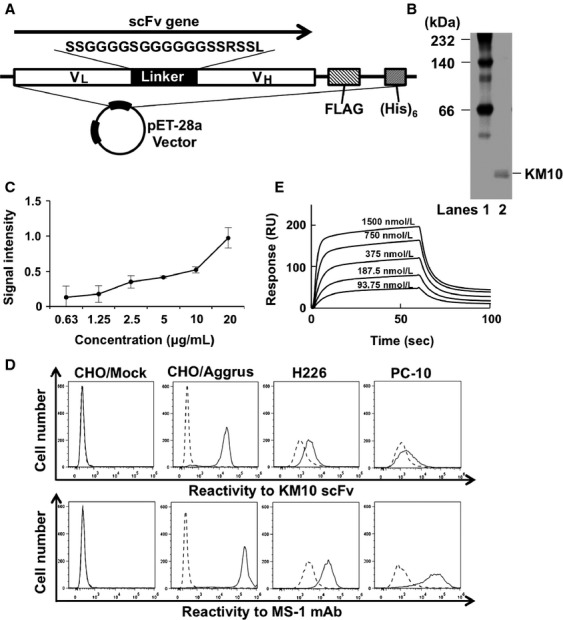
Characterization of KM10 scFv generated from MS-1 mAb. (A) Schematic representation of the generated scFv expression vector. (B) The purity of the used KM10 scFv was analyzed in native PAGE electrophoresis. Lane 1, molecular weight marker; Lane 2, purified KM10 scFv. (C) Bound KM10 scFv in Aggrus-derived P4262 peptide-coated plates were detected using peroxidase-conjugated anti-FLAG antibody. Data are presented as the means ± SDs of triplicate measurements. (D) CHO cells transfected with empty vectors (CHO/Mock) or Aggrus expression plasmid (CHO/Aggrus), as well as H226 and PC-10 cells were treated with 5 *μ*g/mL of KM10 scFv (upper panels) or MS-1 mAb (lower panels). The cells were then treated with Alexa Fluor 488-conjugated secondary antibody. The broken lines represent the cells treated with secondary antibody alone and the bold lines represent KM10 scFv- or MS-1 mAb-treated cells. (E) The indicated five concentrations of KM10 scFv were passed over the chips containing immobilized recombinant human Aggrus-Fc protein using the Biacore X100 system for protein interaction analysis.

The V_H_ and V_L_ domains of mouse mAb MS-1 [10] were cloned and joined together in a V_L_–V_H_ orientation using a peptide linker with appropriate primers (shown in Fig. 1A). A fusion (FLAG) tag was ligated to the C terminal of scFv to ensure detection. The generated KM10 scFv gene was then subcloned into a pET-28a vector (Novagen, Madison, WI) in frame with a (His)_6_ tag sequence to ensure purification. The affinity maturated K-11 scFv gene was accomplished using the QuickChange site-directed mutagenesis kit (Stratagene, La Jolla, CA) using KM10 scFv in pET-28a vector as a template.

### Expression and purification of scFv

BL21 (DE3) *E. coli* competent cells (Life Technologies, Carlsbad, CA) transformed with a pET-28a vector containing KM10 or K-11 scFv were grown in lysogeny broth at 37°C. scFv expression was induced by adding 1 mmol/L isopropyl-*β*-D(−)-thiogalactopyranoside (Wako) and incubated at 37°C for 5 h. After sonication for 15 min, the pellet was solubilized in 50 mmol/L Tris-HCl (pH 7.6) containing 6 mol/L guanidine-HCl and 10 mmol/L 2-Mercaptoethanol at 4°C. The solubilized scFv was purified using Ni-NTA agarose (Qiagen, Venlo, Netherlands). The purified scFv was reduced by adding 10 mmol/L 2-mercaptoethanol for 2 h at room temperature. Furthermore, the denatured 7.5 *μ*mol/L scFv was refolded according to the method by Tsumoto et al. [18] The refolded scFv was purified by size exclusion chromatography on a HiLoad 16/60 superdex 75 prep grade column (GE Healthcare, Buckinghamshire, UK).

### Enzyme-linked solvent assay

To confirm the binding capability of the generated scFv, we firstly purified Aggrus-derived P4262 peptide, that consists of amino acids 42–62 of human Aggrus [10]. As reported previously [10], we used tandemly connected P4262 peptide as an immunogen to generate Aggrus-neutralizing MS-1 mAb. In this study, the purified tandemly connected P4262 peptide was immobilized on the amino-type enzyme-linked immunosorbent assay (ELISA) plates (Sumitomo Bakelite, Tokyo, Japan). After blocking, the plates were incubated with scFv, and then further incubated with peroxidase-conjugated anti-FLAG tag antibody (Sigma-Aldrich, St. Louis, MO), followed by the addition of 1-Step Ultra TMB-ELISA reagent (Thermo Fisher scientific, Waltham, MA).

### Flow cytometric analysis

Cells were harvested and treated with KM10 scFv, K-11 scFv, MS-1 mAb, and D2-40 mAb (AbD Serotec, Oxfordshire, UK), followed by incubation with Alexa Fluor 488-conjugated second antibody.

### Surface plasmon resonance analysis

Surface plasmon resonance (SPR) analysis was performed using Biacore X100 system (GE Healthcare). The Aggrus-Fc was immobilized on a CM5-senser chip (GE Healthcare) according to the manufacturer's protocol [10]. Final levels of immobilization were approximately 500 response units. Five concentrations of KM10 or K-11 scFv were passed over the chip. The equilibrium dissociation constant (*K*_*D*_) was determined using Biacore X100 evaluation software.

### Construction of mutation library

Random mutagenesis was introduced into the KM10 gene using Diversify PCR random mutagenesis kit (TaKaRa BIO, Shiga, Japan). Mutagenesis was performed under the condition of 4.6 mutations per 1000 base pairs. PCR products were subcloned into the phagemid vector pCANTAB 5E. After transformation into XL-1 Blue competent *E. coli* cells, phages displaying randomly mutated KM10 scFv were rescued by the addition of 5 × 10^10^ plaque-forming unit (pfu) of M13KO7 helper phage (New England Biolabs, Hertfordshire, UK). After filtration, the phages were recovered from the supernatant by precipitation with 1/5 volume of 200 *μ*g/mL of polyethylene glycol and 146.1 mg/mL of NaCl. The precipitated phages were resuspended in NTE buffer (100 mmol/L NaCl, 10 mmol/L Tris (pH 7.5), and 1 mmol/L ethylenediaminetetraacetic acid) and stored at −80°C in 15% glycerol until use.

### Panning

For the first, second, and third round of panning, 1.0, 0.5, and 0.1 *μ*g/mL of human Aggrus-derived P4262 peptide were immobilized on a 96-well plate, respectively. After blocking, the phage library was added to the wells containing the immobilized peptides. After shaking for 60 min, the plates were washed 20 times with phosphate-buffered saline (PBS) containing 0.1% Tween 20 and further washed 20 times with PBS. Phages bound to the immobilized P4262 peptides were released using 0.1 mol/L glycine-HCl (pH 2.2) and neutralized by adding 1 mol/L Tris-HCl (pH 9.1). Furthermore, the XL-1 Blue competent *E. coli* cells were infected with the neutralized eluate, and the phages were rescued and collected as described in previous section. After panning three times, the phage library was reinfected into XL1-Blue cells and then plated on YTAG medium plates to obtain single colonies. Individual colonies were propagated, purified, and sequenced. In some experiments, 4 × 10^8^ pfu/mL of phages displaying mutated scFv were added to P4262 peptide-coated plates, which were then incubated with peroxidase-conjugated anti-M13 phage mAb (GE Healthcare).

### Platelet aggregation assay

CHO/Aggrus cells (2 × 10^7^ cells/mL) [10] were incubated with 10 *μ*g/mL of KM10 scFv or K-11 scFv for 30 min on ice. Platelet aggregation assay was performed using washed murine platelets as previously described [19].

### Animals

Female BALB/c-*nu/nu* mice were purchased from Charles River Laboratories Japan, Inc. (Kanagawa, Japan). Jcl:ICR mice were purchased from Clea Japan, Inc. (Tokyo, Japan). All animal procedures were performed according to protocols approved by the Japanese Foundation for Cancer Research Animal Care and Use Committee.

### Ex vivo imaging of lung retention

CHO/Aggrus cells were incubated with CellTrace calcein green AM (calcein-AM; Life Technologies) at 37°C for 30 min. Calcein-AM-labeled CHO/Aggrus cells were incubated with 50 *μ*g/mL of K-11 scFv in Hanks' balanced salt solutions (HBSS; Gibco, Carlsbad, CA) on ice. After incubation for 30 min, 200 *μ*l of the cell suspension (1 × 10^5^ cells) was intravenously inoculated into the lateral tail vein of 8-week-old female BALB/c-*nu/nu* mice. After 1 h of tumor inoculation, frozen sections of lung tissue were prepared, and the number of calcein-AM-labeled micro metastatic foci was counted in two independent view fields for each mouse (a total of four view fields). Nuclei were stained with Hoechst 33342 (Life Technologies).

### Experimental lung metastasis

CHO/Aggrus cells were harvested (2 × 10^6^ cells/mL) and suspended in HBSS. After incubation with PBS or 150 *μ*g/mL of K-11 scFv for 30 min on ice, 200 *μ*L of the cell suspension (2 × 10^5^ cells) was intravenously inoculated into the lateral tail vein of 8-week-old female BALB/c-*nu/nu* mice. After 18 days of tumor inoculation, the lungs were extracted from each mouse and surface metastatic foci were counted.

## Results

### Generation of scFv from neutralizing anti-human Aggrus mAb MS-1

To generate KM10 scFv specific for human Aggrus, the V_H_ and V_L_ domains of mouse mAb MS-1 [10] together with a peptide linker were subcloned into a pET-28a vector (Fig. 1A). KM10 scFv expressed in *E. coli* was purified, refolded, and electrophoresed (Fig. 1B). Using the purified KM10 scFv, we analyzed its binding to the human Aggrus-derived P4262 peptide, which had been used as an immunogen to generate MS-1 mAb [10]. As shown in Figure 1C, purified KM10 scFv bound to the immobilized P4262 peptide in a dose-dependent manner. We further examined the reactivity of KM10 scFv to Aggrus protein using CHO cells that had been transfected with Aggrus-expressing plasmids (CHO/Aggrus), H226, and PC-10 cells [10]. As shown in Figure 1D (upper panels), KM10 scFv bound to the Aggrus-positive CHO/Aggrus, H226, and PC-10 cells but not to the mock-transfected CHO cells (CHO/Mock). Aggrus expression in all of the cell lines, with the exception of the CHO/mock cells, was confirmed using MS-1 mAb (Fig. 1D, bottom panels). Furthermore, we estimated the specificity of KM10 scFv. As previously reported, G45A mutation in human Aggrus abolished MS-1 mAb reactivity and D49A mutation attenuated recognition by MS-1 mAb (Fig. S1, middle panel). The reactivity of KM10 scFv coincided with that of MS-1 mAb, although the total expression levels of wild-type and point-mutated Aggrus estimated by D2-40 mAb appeared to be equal (Fig. S1, top and bottom panels). These results suggest that KM10 scFv could recognize the same epitope of MS-1 mAb. SPR analysis revealed that KM10 scFv bound to immobilized human Aggrus protein with a *K*_*D*_ value of 406.2 nmol/L (Fig. 1E). Because the *K*_*D*_ value of MS-1 mAb was approximately 9 nmol/L [10], the affinity of the generated KM10 scFv was decreased to approximately one forty-fifth.

### Affinity maturation by phage display technology

**Figure 2:**
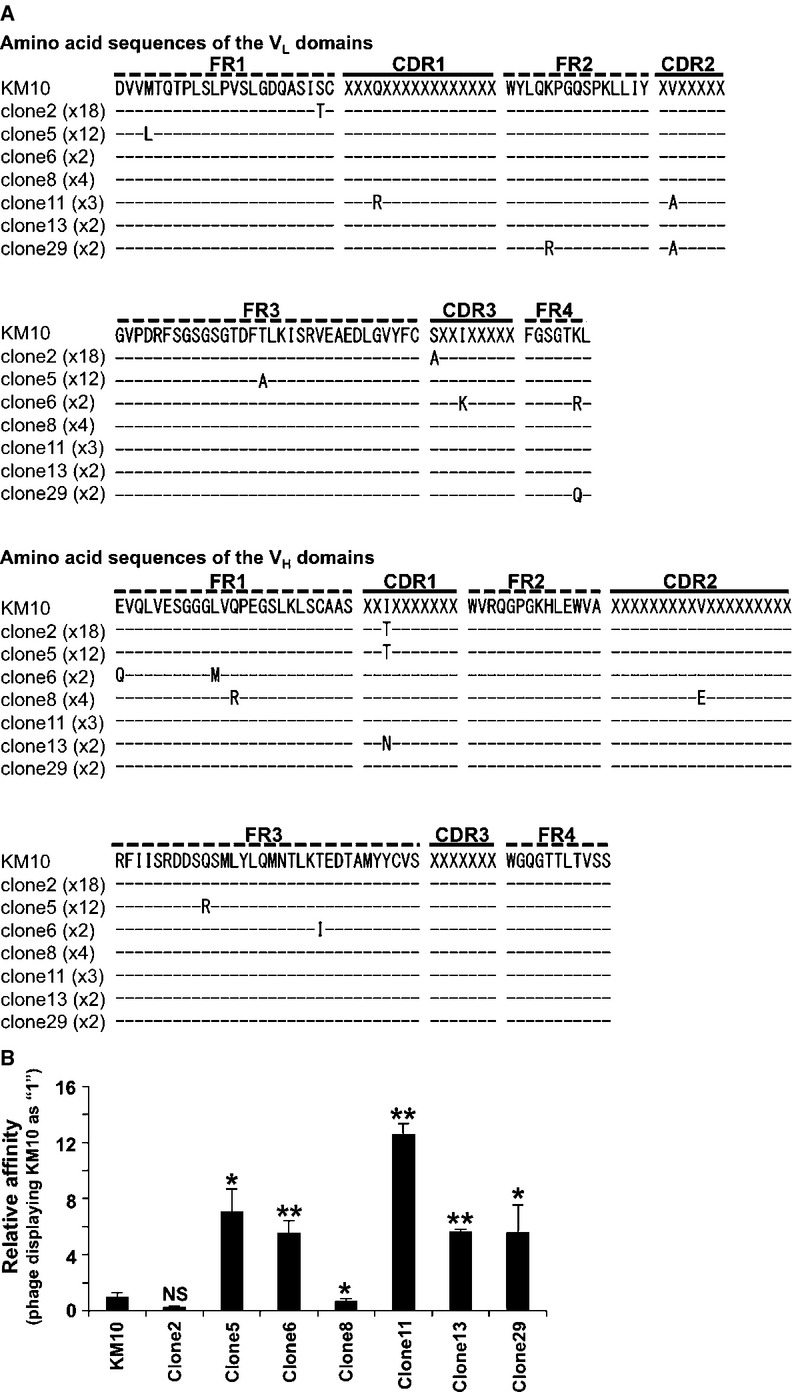
Affinity maturation by phage display technology. (A) Amino acid sequences of the obtained phages are described in single-letter code. Amino acids that matched with KM10 scFv sequence are represented by dash. The number of obtained phage colonies was described in parentheses. CDR, complementarity-determining region; FR, framework region. (B) Phages displaying mutated scFv were added to plate coated with human Aggrus-derived P4262 peptide and then incubated with peroxidase-conjugated anti-M13 phage mAb. Data are presented as means ± SDs of triplicate measurements. NS, not significant. *P < 0.05 or **P < 0.01 by the Student's *t*-test.

scFv can be displayed on the phage surfaces as a functional protein that retains an active antigen-binding domain [20]. In order to improve the affinity of KM10 scFv, we performed affinity maturation by phage display technology using human Aggrus-derived P4262 peptide-coating plate. A mutated sublibrary was created by introducing random mutations in the KM10 scFv gene. Approximately three nucleoic acid mutations were introduced per scFv gene (data not shown). Furthermore, we performed three rounds of panning using a phage library containing randomly mutated KM10 scFv gene and obtained several phage-infected colonies of which 62 were chosen for nucleic acid sequence analysis. Of these, positive sequences could not be obtained from five phages; thus the rest 57 phages were chosen for analysis. We identified 7 patterns of amino acid mutations that were observed in more than 2 independent phages (Fig. 2A). In addition, we found a phage harboring no amino acid mutations and 13 patterns of amino acid mutations that were independently detected from 13 phages. We focused on the former 7 patterns of amino acid mutations because the same amino acid mutations were detected in more than 2 independent phages (Fig. 2A). We first analyzed the binding reactivity of the scFv-expressing phages using ELISA. The reactivity of clone11 phage was the highest among the obtained phages (Fig. 2B). Clone11 phage had mutations in the first and second complementarity-determining region (CDR1 and CDR2, respectively) of the light chain (Q27R and V56A, respectively). We also observed that clone29 phage, which harbored a mutation at Val^56^ in CDR2 of the light chain, showed increase in reactivity to the human Aggrus-derived P4262 peptide (Fig. 2B), though clone29 phage contained additional amino acid mutations in framework regions that would not contribute to the antigen recognition [21]. These results suggest that these two amino acid mutations (Q27R and V56A) additively contributed to the improvement in KM10 scFv affinity to Aggrus protein.

### K-11 scFv suppressed Aggrus-induced platelet aggregation

**Figure 3:**
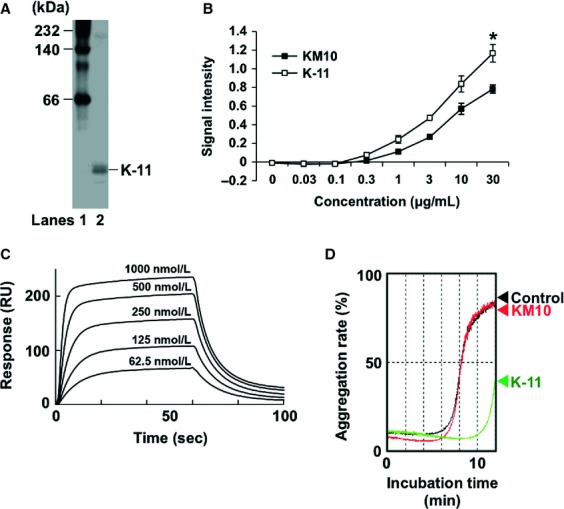
Effect of KM10 and K-11 scFvs on Aggrus-induced platelet aggregation. (A) The purity of the used K-11 scFv was analyzed in native PAGE electrophoresis. Lane 1, molecular weight marker; Lane 2, purified K-11 scFv. (B) Bound KM10 and K-11 scFvs in P4262 peptide-coated plates were detected using peroxidase-conjugated anti-FLAG antibody. Data are presented as means ± SDs of triplicate measurements. *P < 0.05 by Student's *t*-test. (C) The indicated five concentrations of K-11 scFv were passed over the chip containing immobilized recombinant human Aggrus-Fc protein and monitored using the Biacore X100 system. (D) CHO/Aggrus cells were preincubated with 10 *μ*g/mL of BSA (black line), KM10 scFv (red line), or K-11 scFv (green line), and then incubated with washed mouse platelets. Light transmittance of each sample was measured as the aggregation rate.

To confirm the increase in antigen recognition capability by the amino acid mutations, we introduced the mutations observed in clone11 into the KM10 scFv gene. After purification of the generated K-11 scFv (Fig. 3A), we examined its reactivity to the immobilized human Aggrus-derived P4262 peptide. As shown in Figure 3B, the recognition capability of K-11 scFv to the P4262 peptide was greatly improved. SPR analysis confirmed the increase in the affinity of K-11 scFv at 2 timepoints with a *K*_*D*_ value of 192.1 nmol/L (Fig. 3C) when compared with the affinity of KM10 scFv (*K*_*D*_ = 406.2 nmol/L).

To analyze the function of the generated scFv, we investigated the inhibitory effects of scFv on Aggrus-induced platelet aggregation. As shown in Figure 3D, K-11 scFv, but not KM10 scFv, was able to attenuate Aggrus-induced platelet aggregation. These results suggest that affinity-maturated K-11 scFv is suitable as an Aggrus inhibitor.

### K-11 scFv suppressed Aggrus-mediated pulmonary metastasis

**Figure 4:**
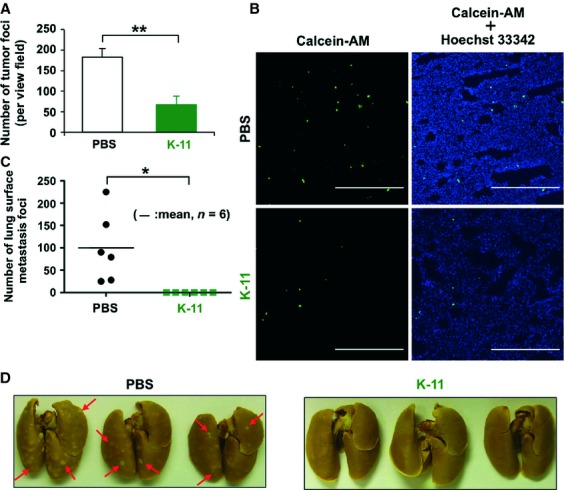
Suppression of Aggrus-induced tumor metastasis by K-11 scFv. (A) and (B) Calcein-AM-labeled CHO/Aggrus cells were preincubated with PBS alone or 50 *μ*g/mL of K-11 scFv on ice for 30 min. The cells were then intravenously inoculated into mice (1 × 10^5^ cells/mouse). After 1 h of tumor inoculation, frozen sections of lung tissues were prepared and the number of calcein-AM-labeled trapped CHO/Aggrus cells was counted in 2 independent fields of view for every two mice (a total of 4 fields of view) (A). **P < 0.01 by Student's *t*-test. Representative pictures of the frozen section of lung are shown (B). Scale bar, 500 *μ*m. (C) and (D) CHO/Aggrus cells were preincubated with 150 *μ*g/mL of K-11 scFv on ice for 30 min and then intravenously inoculated into BALB/c-*nu/nu* mice (2 × 10^5^ cells/mouse). After 18 days of tumor inoculation, metastatic foci on the lung surface were counted. Numbers of metastatic foci in each mouse are shown (C). Bars, mean (*n* = 6). *P < 0.05 by the Mann–Whitney *U*-test. Representative pictures of the lungs and lung surface metastatic foci (indicated by red arrows) are shown (D).

Because K-11 scFv, but not KM10 scFv, attenuated Aggrus-induced platelet aggregation in vitro, we then examined the inhibitory effect of K-11 scFv on pulmonary metastasis in vivo. When calcein-AM-labeled CHO/Aggrus cells were intravenously injected into mice, we were able to detect trapped CHO/Aggrus cells in the lung tissue under fluorescent microscopy (Figs. 4A and B). Using this method, we investigated the inhibitory effects of K-11 scFv on lung retention of CHO/Aggrus cells in vivo. After incubating CHO/Aggrus cells with 50 *μ*g/mL of K-11 scFv or buffer alone (PBS), the cell suspension was intravenously injected into nude mice. After 1 h of tumor inoculation, the fluorescent cells in lung microvessels were counted. As shown in Figure 4A and B, treatment of CHO/Aggrus cells with K-11 scFv significantly decreased the number of cells trapped in the lungs. These results encouraged us to examine the metastasis inhibitory effects of scFv in vivo. As expected, preincubation of CHO/Aggrus cells with 150 *μ*g/mL of K-11 scFv significantly suppressed the number of lung surface metastasis foci (Figs. 4C and D). These results indicate that our generated anti-Aggrus K-11 scFv may be useful to prevent pulmonary metastasis of Aggrus-positive tumor cells in vivo.

## Discussion

We first attempted to generate scFv with a V_H_-V_L_ orientation by linking V_H_ and V_L_ with a peptide linker. However, scFv expressed in *E. coli* formed aggregates that were present in the inclusion bodies. Although Zheng et al. [22] reported that they succeeded in purifying active scFv by adding a Nus tag to the N terminal, which reportedly greatly enhances the solubility of fusion proteins, we detected no scFv in soluble fraction. Therefore, we recovered functional scFv from inclusion bodies using the method reported by Tsumoto et al. [18]. However, the purified scFv failed to bind to Aggrus (data not shown). The orientation of V_H_ and V_L_ (i.e., V_H_-V_L_ or V_L_-V_H_) in scFv is known to affect its antigen-binding activity [17]. Hence it is possible that the V_H_-V_L_ orientation scFv may be inappropriate for use in Aggrus-binding investigations. Thus, we generated KM10 scFv in the V_L_–V_H_ orientation and observed that the purified KM10 scFv recognized Aggrus, approximating that of MS-1 mAb.

Conversion of IgG to scFv often results in the loss of its affinity [17]. In our experiment, we observed a 45-fold decrease in the affinity of KM10 scFv (Fig. 1E). Phage display technology is the most frequently used method to improve the affinity of scFv because scFv can be displayed on the phage surfaces as a functional protein and retains a functional antigen-binding domain [20]. Using phage display technology, we succeeded to obtain K-11 scFv exhibiting increased antigen recognition capability and decreased *K*_*D*_ value than KM10 scFv. In this screening, we selected phages bound to P4262 peptide (amino acids 42–62 of human Aggrus). Because we generated Aggrus neutralizing MS-1 mAb by immunizing P4262 peptide, MS-1 mAb-derived KM10 scFv would recognize the same epitope containing in P4262 peptide that is essential for exhibiting Aggrus neutralizing activity. This is the reason why we used P4262 peptide, but not Aggrus protein, for screening to exclude the possibility that the obtained scFv mutants could not exhibit neutralizing activity due to the change in recognizing epitopes. We then compared K-11 scFv with KM10 scFv with FACS analysis (Fig. S2). In FACS analysis, although the effect on CHO/Aggrus is little difference from KM10 scFv to K-11 scFv, the binding capability of K-11 scFv to CHO/Aggrus-D49A was markedly elevated (Figs. S1 top panel and S2). These results show that phage display screening restored the altered epitope recognition capability of KM10 scFv to that of MS-1 mAb. Recently it is problem that tumor cells acquire resistant on molecular target drugs by replacing drug recognition site or drug-binding site with other amino acid. In this study, the affinity-maturated K-11 scFv could also bind to CHO/Aggrus-D49A. These results suggested that K-11 scFv might overcome resistant problem caused by D49A amino acid replacement. Moreover, by using phage display technology, although the affinity of K-11 scFv was increased only twofold compared with that of KM10 scFv (Fig. 3C), Pavoni et al. [23] successfully increased the affinity of anti-CEA scFv by more than 10-fold after panning for a second time consisting of three consecutive rounds. The first round of panning introduced random mutations into the heavy and light chains of CDR3, which is the region most important for antigen–antibody reactions, and the second round introduced others. Yang et al. [24] reported that the affinity scFv was increased by a few 100-fold following random mutagenesis in only four CDRs (CDR1 or 3 of the heavy and light chains, respectively). After 5–10 rounds of panning, combining each mutation site increased affinity. In the present study, we achieved improved affinity of K-11 scFv after three rounds of panning. In future experiments, we plan to perform several rounds of panning to increase affinity of scFv.

Although Chandramohan et al. [25] recently reported antitumor activity of scFv targeting Aggrus/podoplanin fused with immunotoxin in an intracranial medulloblastoma model using scFv to target the tumor, Aggrus-neutralizing activity of it was not examined. Thus, this is the first report to generate scFv that retained Aggrus-neutralizing activity. Because the platelet aggregation-inducing ability of Aggrus is essential for tumor metastasis-promoting ability [5], it is not surprising that platelet aggregation-neutralizing K-11 scFv was observed to suppress pulmonary metastasis in vivo. We recently observed that the growth of some tumor cells was promoted by platelet-secreted growth factors [10, 26]. Therefore, it is possible that K-11 scFv exhibits antitumor activity against Aggrus-positive tumors by inhibiting Aggrus-mediated platelet aggregation. Hence the antitumor activity of K-11 scFv warrants further investigations.

In general, scFv is preferred than whole antibody because of its smaller size that enables tumor penetration, its lesser possibility to develop HAMA (human anti-mouse antibody) response, and its higher blood clearance that reduces side effects [16]. Moreover, scFv can be easily fused to fluorescent proteins, radionuclides, or cytotoxic drugs for therapeutic and diagnostic purposes. These advantages would also apply to our generated K-11 scFv. We will continue the development of K-11 scFv as a new tool to aid in the development of Aggrus-targeted metastasis inhibitors.

## Supporting Information

**Figure S1.** Characterization of the KM10 scFv generated from MS-1 mAb. CHO cells that had been transfected with WT-, G45A-, or D49A-Aggrus expression plasmids (CHO/Aggrus-WT, CHO/Aggrus-G45A, or CHO/Aggrus-D49A, respectively) or with empty vector (CHO/Mock) were treated with 10 *μ*g/mL of KM10 scFv (top panels), MS-1 mAb (middle panels), or D2-40 mAb (bottom panels). The cells then were treated with Alexa Fluor 488-conjugated anti-FLAG (for KM10 scFv) or anti-mouse IgG (for MS-1 or D2-40 mAb) antibody (bold lines). The broken lines represent the cells treated with secondary antibody alone.

**Figure S2.** Characterization of the K-11 scFv. CHO cells that had been transfected with WT-, G45A-, or D49A-Aggrus expression plasmids (CHO/Aggrus-WT, CHO/Aggrus-G45A, or CHO/Aggrus-D49A, respectively) or with empty vector (CHO/Mock) were treated with 10 *μ*g/mL of K-11 scFv. The cells then were treated with Alexa Fluor 488-conjugated anti-FLAG antibody (bold lines). The broken lines represent the cells treated with secondary antibody alone.

## References

[1] Chaffer CL, Weinberg RA (2011). A perspective on cancer cell metastasis. Science.

[2] Gay LJ, Felding-Habermann B (2011). Contribution of platelets to tumour metastasis. Nat. Rev. Cancer.

[3] Honn KV, Tang DG, Crissman JD (1992). Platelets and cancer metastasis: a causal relationship?. Cancer Metastasis Rev.

[4] Ruggeri ZM, Mendolicchio GL (2007). Adhesion mechanisms in platelet function. Circ. Res.

[5] Fujita N, Takagi S (2012). The impact of Aggrus/podoplanin on platelet aggregation and tumour metastasis. J. Biochem.

[6] Klerk CP, Smorenburg SM, Otten HM, Lensing AW, Prins MH, Piovella F (2005). The effect of low molecular weight heparin on survival in patients with advanced malignancy. J. Clin. Oncol.

[7] Kato Y, Fujita N, Kunita A, Sato S, Kaneko M, Osawa M (2003). Molecular identification of Aggrus/T1alpha as a platelet aggregation-inducing factor expressed in colorectal tumors. J. Biol. Chem.

[8] Raica M, Cimpean AM, Ribatti D (2008). The role of podoplanin in tumor progression and metastasis. Anticancer Res.

[9] Yuan P, Temam S, El-Naggar A, Zhou X, Liu DD, Lee JJ (2006). Overexpression of podoplanin in oral cancer and its association with poor clinical outcome. Cancer.

[10] Takagi S, Sato S, Oh-hara T, Takami M, Koike S, Mishima Y (2013). Platelets promote tumor growth and metastasis via direct interaction between Aggrus/podoplanin and CLEC-2. PLoS ONE.

[11] Holliger P, Hudson PJ (2005). Engineered antibody fragments and the rise of single domains. Nat. Biotechnol.

[12] Sliwkowski MX, Mellman I (2013). Antibody therapeutics in cancer. Science.

[13] Carter PJ (2006). Potent antibody therapeutics by design. Nat. Rev. Immunol.

[14] Scott CT (2005). The problem with potency. Nat. Biotechnol.

[15] Chadd HE, Chamow SM (2001). Therapeutic antibody expression technology. Curr. Opin. Biotechnol.

[16] Ahmad ZA, Yeap SK, Ali AM, Ho WY, Alitheen NB, Hamid M (2012). scFv antibody: principles and clinical application. Clin. Dev. Immunol.

[17] Weisser NE, Hall JC (2009). Applications of single-chain variable fragment antibodies in therapeutics and diagnostics. Biotechnol. Adv.

[18] Tsumoto K, Shinoki K, Kondo H, Uchikawa M, Juji T, Kumagai I (1998). Highly efficient recovery of functional single-chain Fv fragments from inclusion bodies overexpressed in Escherichia coli by controlled introduction of oxidizing reagent–application to a human single-chain Fv fragment. J. Immunol. Methods.

[19] Nakazawa Y, Takagi S, Sato S, Oh-hara T, Koike S, Takami M (2011). Prevention of hematogenous metastasis by neutralizing mice and its chimeric anti-Aggrus/podoplanin antibodies. Cancer Sci.

[20] McCafferty J, Griffiths AD, Winter G, Chiswell DJ (1990). Phage antibodies: filamentous phage displaying antibody variable domains. Nature.

[21] Wilson IA, Stanfield RL (1994). Antibody-antigen interactions: new structures and new conformational changes. Curr. Opin. Struct. Biol.

[22] Zheng L, Baumann U, Reymond JL (2003). Production of a functional catalytic antibody ScFv-NusA fusion protein in bacterial cytoplasm. J. Biochem.

[23] Pavoni E, Flego M, Dupuis ML, Barca S, Petronzelli F, Anastasi AM (2006). Selection, affinity maturation, and characterization of a human scFv antibody against CEA protein. BMC Cancer.

[24] Yang WP, Green K, Pinz-Sweeney S, Briones AT, Burton DR, Barbas CF (1995). CDR walking mutagenesis for the affinity maturation of a potent human anti-HIV-1 antibody into the picomolar range. J. Mol. Biol.

[25] Chandramohan V, Bao X, Kato-Kaneko M, Kato Y, Keir ST, Szafranski SE (2013). Recombinant anti-podoplanin (NZ-1) immunotoxin for the treatment of malignant brain tumors. Int. J. Cancer.

[26] Takagi S, Takemoto A, Takami M, Oh-hara T, Fujita N (2014). Platelets promote osteosarcoma cell growth through activation of the platelet-derived growth factor receptor-Akt signaling axis. Cancer Sci.

