# Supplementary figures and images for: Suppression of Aggrus/podoplanin-induced platelet aggregation and pulmonary metastasis by a single-chain antibody variable region fragment
Source: Cancer Med. 2014 Aug 16;3(6):1595–604. doi: 10.1002/cam4.320 (PMC4298386; doi:10.1002/cam4.320)

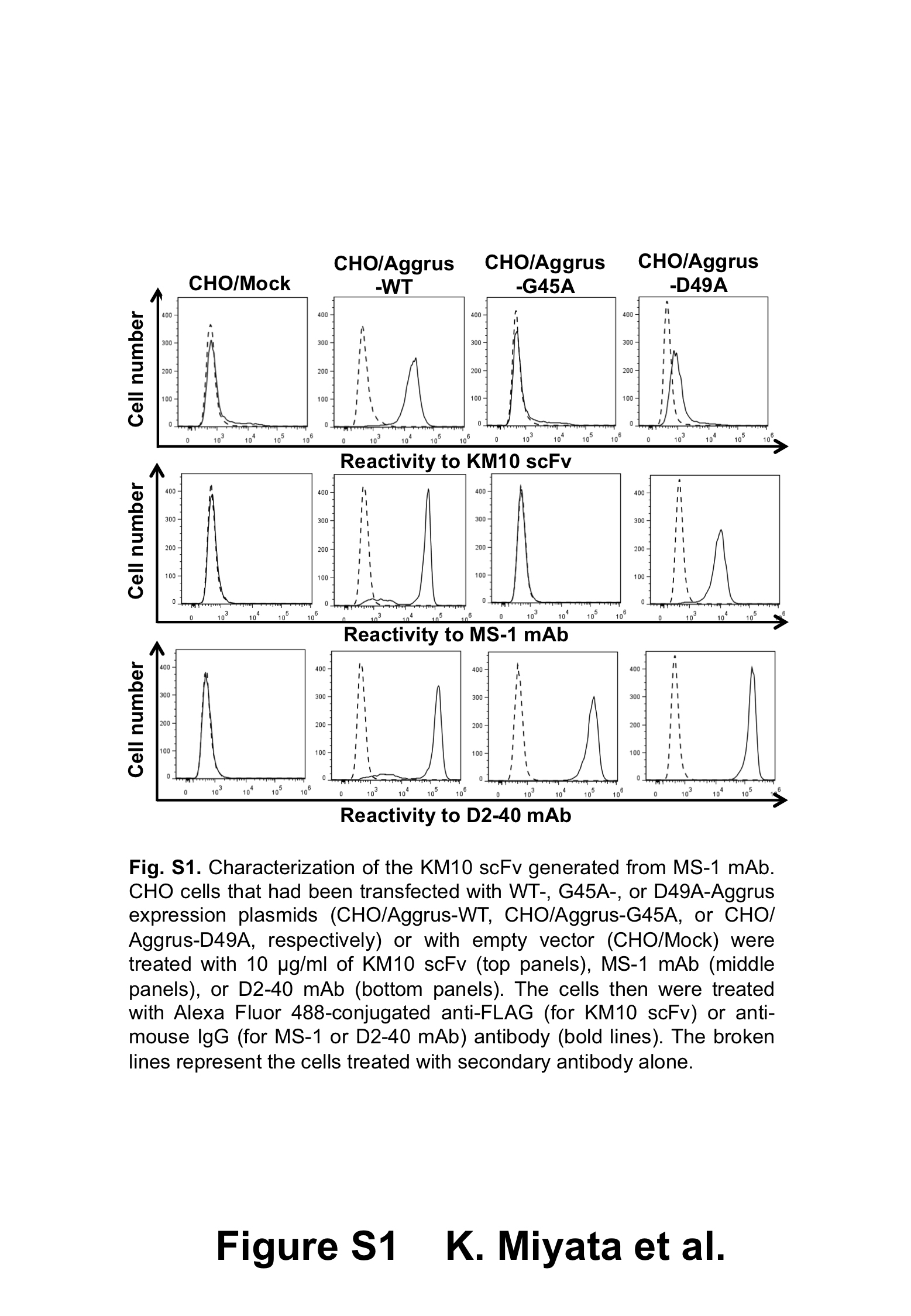

Supplement: Supplementary file 1 — Figure S1. Characterization of the KM10 scFv generated from MS-1 mAb. CHO cells that had been transfected with WT-, G45A-, or D49A-Aggrus expression plasmids (CHO/Aggrus-WT, CHO/Aggrus-G45A, or CHO/Aggrus-D49A, respectively) or with empty vector (CHO/Mock) were treated with 10 μg/mL of KM10 scFv (top panels), MS-1 mAb (middle panels), or D2-40 mAb (bottom panels). The cells then were treated with Alexa Fluor 488-conjugated anti-FLAG (for KM10 scFv) or anti-mouse IgG (for MS-1 or D2-40 mAb) antibody (bold lines). The broken lines represent the cells treated with secondary antibody alone. [file cam40003-1595-sd1.jpg]

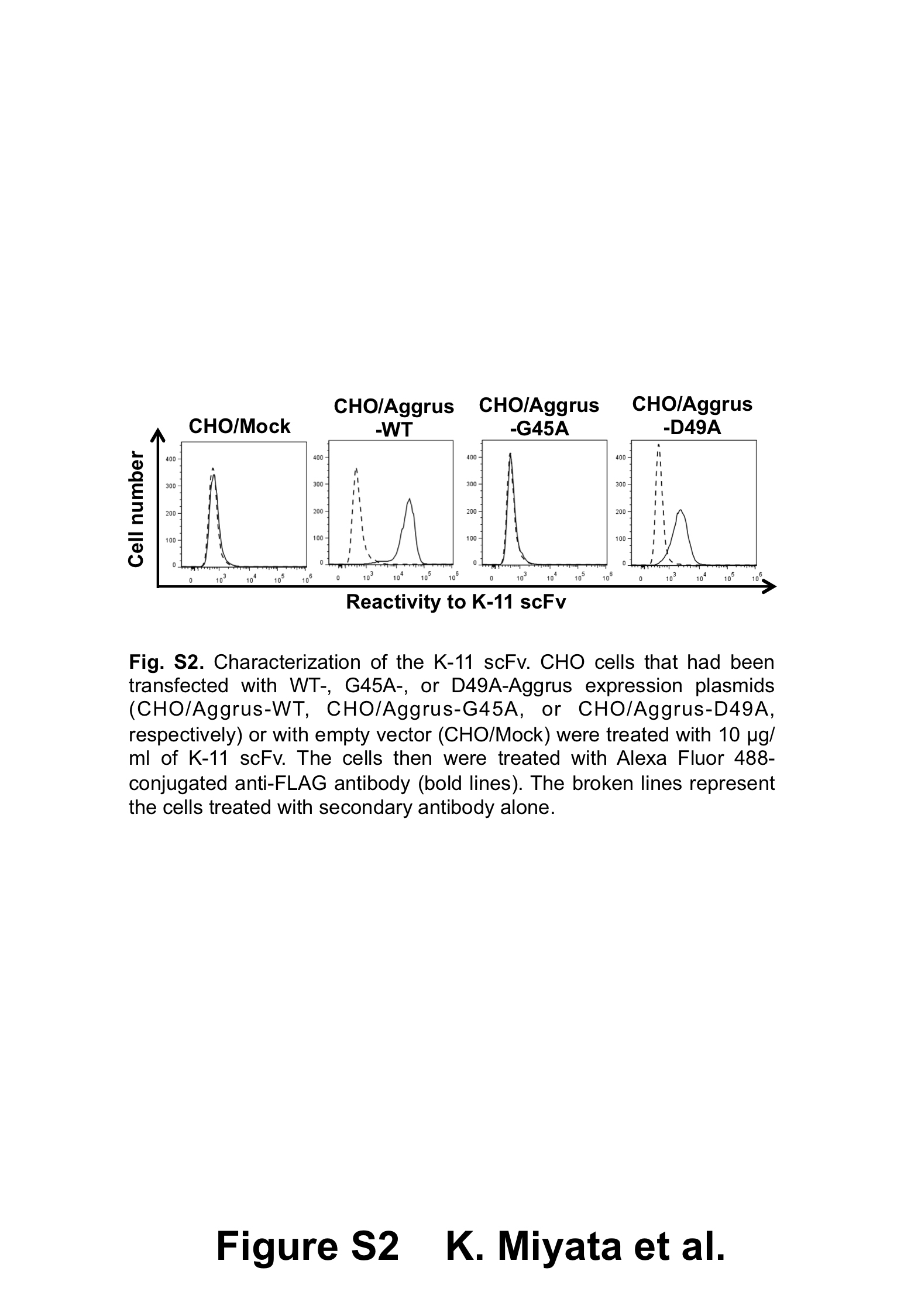

Supplement: Supplementary file 2 — Figure S2. Characterization of the K-11 scFv. CHO cells that had been transfected with WT-, G45A-, or D49A-Aggrus expression plasmids (CHO/Aggrus-WT, CHO/Aggrus-G45A, or CHO/Aggrus-D49A, respectively) or with empty vector (CHO/Mock) were treated with 10 μg/mL of K-11 scFv. The cells then were treated with Alexa Fluor 488-conjugated anti-FLAG antibody (bold lines). The broken lines represent the cells treated with secondary antibody alone. [file cam40003-1595-sd2.jpg]
